# Rapid and Ultrasensitive Sensor for Point-of-Use Detection of Perfluorooctanoic Acid Based on Molecular Imprinted Polymer and AC Electrothermal Effect

**DOI:** 10.3390/mi16030283

**Published:** 2025-02-28

**Authors:** Niloufar Amin, Jiangang Chen, Ngoc Susie Nguyen, Qiang He, John Schwartz, Jie Jayne Wu

**Affiliations:** 1 Department of Electrical Engineering and Computer Science, The University of Tennessee, Knoxville, TN 37996, USA; namin2@utk.edu; 2 Department of Public Health, The University of Tennessee, Knoxville, TN 37996, USA; jchen38@utk.edu; 3 Department of Electrical Engineering and Computer Engineering, California State University, Chico, CA 95929, USA; 4 Department of Civil and Environmental Engineering, The University of Tennessee, Knoxville, TN 37996, USA; qianghe@utk.edu (Q.H.); jschwart@utk.edu (J.S.)

**Keywords:** capacitive sensing, molecularly imprinted polymers, point-of-use sensor, perfluorooctanoic acid, AC electrokinetic, microfluidics

## Abstract

Perfluorooctanoic acid (PFOA) is one of the most persistent and bioaccumulative water contaminants. Sensitive, rapid, and in-field analysis is needed to ensure safe water supplies. Here, we present a single step (one shot) and rapid sensor capable of measuring PFOA at the sub-quadrillion (ppq) level, 4.5 × 10^−4^ ppq, within 10 s. This innovative sensor employs a synergistic combination of a molecularly imprinted polymer (MIP)-modified gold interdigitated microelectrode chip and AC electrothermal effects (ACETs), which enhance detection sensitivity by facilitating the accelerated movement of PFOA molecules towards specific recognition sites on the sensing surface. The application of a predetermined AC signal induces microfluidic enrichment and results in concentration-dependent changes in interfacial capacitance during the binding process. This enables real-time, rapid quantification with exceptional sensitivity. We achieved a linear dynamic range spanning from 0.4 to 40 fg/L (4 × 10^−7^–4 × 10^−5^ ppt) and demonstrated good selectivity (~1:100) against other PFAS compounds, including perfluorooctanoic acid (PFOS), in PBS buffer. The sensor’s straightforward operation, cost-effectiveness, elimination of the need for external redox probes, compact design, and functionality in relatively resistant environmental matrices position it as an outstanding candidate for deployment in practical applications.

## 1. Introduction

Perfluorinated compounds (PFCs) are a group of man-made chemicals that have drawn significant attention because of their environmental persistence and potential adverse effects on human health [1]. The widespread use of PFCs, also known as per- and polyfluorinated alkyl substances (PFASs), in our daily lives such as in household products, fire retardants, and water repellents, along with their resilience against degradation processes, has led to their release into the environment, resulting in the contamination of various soil and water sources [2]. Among the different types of PFASs, per-fluorooctanesulfonic acid (PFOS) and perfluorooctanoic acid (PFOA) are of concern and the most investigated PFASs, owing to their significant environmental prevalence and toxicity [3]. Considering increasing concerns, the US Environmental Protection Agency (EPA) replaced the initial PFAS health advisory limit of 70 ppt (ng/L) for PFOS in combination with PFOA in drinking water to the new enforceable limits of 4 ppt for these compounds [4]. These updated levels act as a warning that even extremely low concentrations of PFOA and PFOS in water could pose potential risks. Thus, the ability to rapidly detect fluctuations in PFOA and PFOS levels in waterways to identify sources of contamination is essential.

Standard chromatographic methods coupled with mass spectrometry for detecting and quantifying PFASs are costly, time consuming, and require transporting samples to central laboratories, making them impractical for routine environmental analysis and continuous monitoring [5,6]. A few instances of PFOA sensing approaches have been reported to reduce costs and simplify the detection process, including fluorescence detection with an aptamer probe [7], mass spectrometry with metal–organic frameworks (MOFs) probe [8], and resonance light scattering detection with a crystal violet probe [9]. However, these sensors either lack sensitivity and selectivity or are not adaptable for field deployment. At this point, a promising alternative probe has emerged to overcome these shortcomings: molecular imprinting polymer (MIP) technology, offering the specificity and selectivity of biological receptors, with the explicit advantages of durability with respect to environmental conditions and a low cost. Molecular imprinting of electrochemically inactive analyte by electropolymerization is well established [10]. Electropolymerization offers precise control over film thickness and morphology, enabling the formation of uniform and reproducible MIP films directly on electrode surfaces. Common electropolymerizable monomers include pyrrole, o-Phenylenediamine (o-PD), aniline, and thiophene derivatives, which facilitate the creation of conducting polymers with imprinted sites complementary to target molecules [11].

Recent studies [12,13,14,15,16,17] using molecularly imprinted polymer (MIP)-based sensors have demonstrated promising analytical performances for PFOA detection. A recent study demonstrated a portable MIP-based microchip and differential pulse voltammetry (DPV) detection method for PFOA with LODs (19 ppt) [12] as well as MIP-based optical fiber sensing device with a very low LOD (0.8 ppt) [16]. Although these studies have leveraged the use of imprinted polymers for PFOA sensing, they are either not adaptable for field deployment, including for the requirement of an external redox probe like for ferrocene carboxylic acid (FcCOOH) [12] and extra wash steps, or lack the sensitivity requirements, struggling to meet the baseline regulatory limits, e.g., 2 × 10^4^ fg/L (0.02 ppt), imposed by the revised EPA guideline [18]. Table 1 provides an overview of various MIP-based PFOA sensors that have been reported.

The MIP matrix forms specific cavities that interact with the template molecule, PFOA, primarily through hydrogen bonds. Upon removing PFOA via solvent extraction, these cavities remain, enabling the MIP to selectively rebind PFOA molecules.

In our previous work, we introduced the application of capacitance-based molecularly imprinted polymer (MIP) sensors for detecting perfluorooctane sulfonate (PFOS) [19]. This study pioneers the detection of PFOA through the innovative application of o-PD as a functional monomer. o-PD is particularly noteworthy due to its amine functionalities, which engage directly with the carboxylate group of PFOA. This research marks the first instance of using o-PD as a functional monomer in the development of molecularly imprinted polymers (MIPs) specifically for PFOA detection.

**Figure 1:**
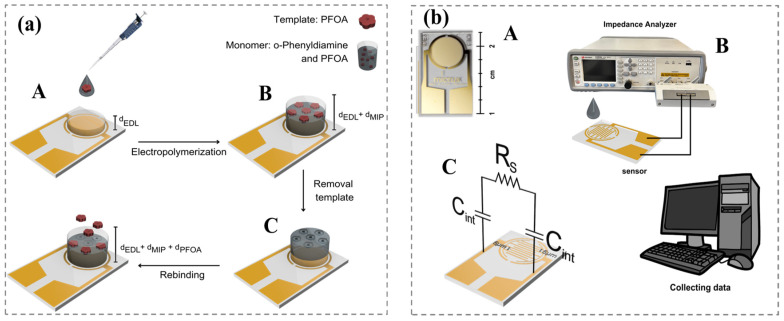
(**a**) A schematic of the PFOA-MIP film electropolymerization on the Au-IDME and a sensing mechanism based on the change in the electrical double layer (EDL). The top transparent layer shows the dielectric layer on the electrode’s surface. (**b**) A photo of a Au-IDME (**A**); the measurement setup for the ACEK-capacitive sensing (**B**); a simplified circuit model employed to represent the electrode–electrolyte interface (**C**). d_EDL_, d_MIP_, and d_PFOA_: the thickness of the EDL, MIP, and PFOA molecules.

By utilizing gold interdigitated microelectrodes (Au-IDMEs) and employing anodic electropolymerization of o-PD in the presence of PFOA, we can precisely control the thickness of the MIP layer that is imprinted with PFOA on the electrode surface. This approach significantly enhances the selectivity of the sensor. Moreover, the integration of the AC electrothermal (ACET) enrichment method and the imprinting polymer sensing layer creates a synergistic effect, significantly enhancing the mass transfer of PFOA to the binding sites within the imprinted cavities. This enhancement improves the sensitivity of detection, shortens the time from sample to result, and makes this sensing system simple to use. Figure 1 provides a visual overview of the sensor preparation, the mechanism of ACEK-capacitive sensing for PFOA, and the measurement system. During the 10 s assay period, an optimized alternating current (AC) signal is applied to gold interdigitated microelectrode (Au-IDME) sensors, which serves two key functions. First, it monitors changes in interfacial capacitance resulting from PFOA deposition on the sensor surface, which is coated with molecularly imprinted polymer (MIP) receptors; second, it enhances the capture of PFOA at the sensor surface through AC electrokinetic (ACET) micro-fluidic effects. This dual functionality is essential for ensuring accurate and efficient detection of PFOA in our sensor system. This represents the first ACEK-capacitive sensor developed with MIP technology for highly sensitive and selective PFOA, offering rapid and user-friendly operation suitable for point-of-use applications.

## 2. Materials and Methods

O-Phenylenediamine (o-PD, ≥98%), perfluorooctanoic acid (PFOA, ≥96%), ferrocenecarboxylic acid (FcCOOH, ≥97%), perfluorooctanesulfonic acid, ammonium buffer solution, octanoic acid, and perfluoroheptanoic acid (PFHA, 99%) were obtained from Sigma-Aldrich. Perfluoropentanoic acid (PFPA, ≥97%) was purchased from Alfa Aasar (Ward Hill, MA, USA). Acetone, isopropyl alcohol (IPA), and phosphate-buffered saline (10×) pH 7.4 were purchased from Thermo Fisher Scientific (Waltham, MA, USA). All the reagents were of analytical grade. Testing buffer 0.1× PBS pH 7.4 was prepared by 1:100 volume dilution of 10× PBS in Milli-Q water. Analytical PFAS samples were prepared as 0.4, 2, 5, 40, and 400 fg/L, in 0.1× PBS. We performed the electropolymerization process with a conventional three-electrode setup with the potentiostat/galvanostat PalmSens 4 with the software PSTrace version 5.9. Thin-film gold interdigitated microelectrodes (r = 4.0 mm) (Ref ED-IDE3-Au) were purchased from Micrux technologies (Asturias, Spain) and used as the working electrode. The electrode fingers are 5 µm wide with 5 µm spacing, comprising 180 pairs. A platinum electrode and an Ag|AgCl|KCl (3M) served as the auxiliary electrodes and the reference electrode, respectively. All electrical measurements were operated using a precision LCR meter (Keysight^®^ E4980A, Santa Rosa, CA, USA).

### 2.1. Fabrication of MIP/Au-IDME

We fabricated the MIP/Au-IDME according to our previous work [19], modifying the protocol originally reported by the Karimian group [20]. The procedures for electrode cleaning and pretreatment by CV are also detailed in our previous work [19]. After surface cleaning, a poly o-PD film was formed on the Au-IDME surface by scanning from 0 to + 1.0 V (vs Ag/AgCl electrode) at 50 mV/s for 25 cycles in 0.1 M acetate buffer, pH 5.8, and methanol (2:1, *v*/*v*) solution containing 10.0 mM o-PD and 1.0 mM PFOA. During the o-PD polymerization process, PFOA template molecules were entrapped in the polymer film. Figure 1a(A–C) demonstrates the schematic fabrication of the MIP/Au-IDME. Au-IDME functionalized with nonimprinted polymer (NIP), which stays in the same condition, but without PFOA addition, serves a dual purpose: (1) acting as a dummy to access the performance of target recognition, and (2) functioning as a control in experimental comparisons. After polymerization, the functionalized electrode was rinsed with milli-Q water and kept in methanol/water (1:1, *v*/*v*) solution for 20 min under mild stirring, using a low-speed magnetic stirrer, to facilitate the removal of the PFOA template, followed by subsequent washing with milliQ water.

### 2.2. Measurement Process and Data Analysis

The functionalized MIP sensor was initially interfaced with an impedance analyzer (Figure 1b(C)), to directly measure and record the interfacial capacitance (C_int_). In the procedure, 10 µL of the target solution was introduced onto the sensing area, followed by the application of an AC signal to the sensor while continuously monitoring the capacitance for a specified 30 s period. This AC signal played a dual role: facilitating capacitance measurement and providing an electric field for PFOA enrichment. According to our previous work [19,21], 3 kHz and 100 mV is proven to be an optimal AC signal for this type of IDME to maximize the ACET effect for molecules of a small size. The sensor response was quantified by the normalized rate of capacitance change, represented as dC/dt (%/min). This was calculated by fitting time-versus-capacitance transient curves using the least-squares method for each dataset.

## 3. Results and Discussion

### 3.1. Molecularly Imprinted Poly o-PD Preparation on Au-IDMEs

**Figure 2:**
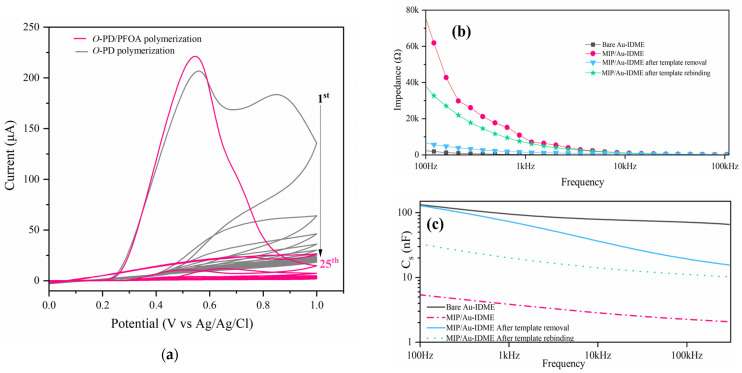
(**a**) Cyclic voltammograms during the electrodeposition of MIP layer on Au-IDME in acetate buffer (pH 5.8) with 10 mM o-PD and 1 mM PFOA (pink line), or 10 mM o-PD only (gray one); scan rate 50 mV/s; number of scans 25. Electrical spectrums for Au-IDME surface characterization. Impedance (**b**) and capacitance (**c**) spectrums before and after MIP electrodeposition and after template.

The electropolymerization of o-PD on the Au-IDME was performed in an acetate buffer solution (Figure 1a). A typical cyclic voltammogram recorded during the electropolymerization of o-PD in the presence of PFOA is shown in Figure 2a. A noticeable decrease in the anodic peak corresponded to irreversible o-PD oxidation on the electrode surface during continuous cycling [19,20]. This illustrates the formation of non-conductive film in the electrode surface suppressing the anodic oxidation of o-PD (Figure 2a).

After polymerization, the PFOA molecules were removed via a washing step, leaving behind cavities with binding sites complementary to the template molecules, as shown in Figure 1a. These specific recognition sites on the MIP film are capable of selectively detecting PFOA (Figure 1a(C)), indicated by a measurable decrease in interfacial capacitance.

### 3.2. Interfacial Capacitance Sensing

When an Au-IDME (Figure 1b(A)) is immersed in an electrolyte, at the interface of a charged electrode surface and electrolyte, where a layer of counter-ions forms to maintain charge neutrality, an electric double layer (EDL) forms [21,22]. The EDL structure can be electrically represented as a parallel-plate capacitor in an electrical equivalent circuit (Figure 1b(C)) [23]. In our previous study [23,24], we optimized buffer solution conditions and frequency range (in 0.1× PBS and within 3 kHz~100 kHz), showing that the impedance response is primarily governed by the interfacial capacitance and electrolyte resistance.

As a result, the circuit model can be further simplified to a series combination of *C_int_* and R_S_ [25], as illustrated in Figure 1b(C). The principle of capacitive sensing relies on detecting changes in surface topology, such as variations in polymer thickness and surface area, which directly influence interfacial capacitance. The capacitance at the interface can be expressed as follows:

(1) $$C_{int}=\frac{A}{\left(\frac{d_{EDL}}{\varepsilon_{s}}+\frac{d_{MIP}}{\varepsilon_{p}}+\frac{d_{PFOA}}{\varepsilon_{t}}\right)}$$

where *d_EDL_*, *d_MIP_*, and *d_PFOA_* are the thickness of the EDL, the capture MIP layer, and PFOA, respectively. *A* is the effective sensor surface area, and *ε_s_*, *ε_p_*, and *ε_t_* are the permitivities of the EDL, capture MIP probes, and PFOA target, respectively.

Figure 1a provides a conceptual overview of the topological changes in the IDE during various stages of sensor preparation and detection. The initial interfacial capacitance consists of the EDL and MIP layer. Upon the capture of PFOA molecules by the MIP, the dielectric layer will become thicker, leading to a decrease in interfacial capacitance (Equation (1)).

### 3.3. PFOA Enrichment by ACET Effect

As we discussed earlier, for analyzing PFAS compounds, the preconcentration is always crucial [6,19]. On the other hand, in the process of detection, target molecules tend to diffuse to the electrode surface and bind with the immobilized probes. The random nature of passive diffusion is the underlying cause for low sensitivity and long testing times [26].

In a nonuniform AC electrical field, AC electrokinetic (ACEK) effects, such as dielectrophoresis, AC electroosmosis and ACET effect, will be stimulated on the Au-IDME in an electrolyte [27]. Among them, ACET effect arising from induced temperature gradients is widely known as a powerful effect used to drive and enrich small molecules such as for PFASs [24,27] in a practical solution such as surface waters. The microflows driven by the conductivity variation will convect small molecules/particles, here PFOA, towards the electrode surface for binding [21,23,24]. Unlike traditional methods requiring extra equipment or long incubation, ACET can rapidly enrich PFAS molecules without additional processes [19]. The ACET velocity *u_ACET_*, at room temperature, can be expressed as follows [25,28]:

(2) $$u_{ACET}=5\times 10^{-4}(\frac{1}{\sigma }\frac{\partial \sigma }{\partial T})\frac{\varepsilon \sigma }{k\eta }\frac{V^{4}}{r}$$

*k*, *σ*, *η*, and *ε* represent the thermal conductivity, electrical conductivity, viscosity, and permittivity of the medium, respectively. *V* denotes the value of the AC voltage. *r* was half of the electrode separation, set at 2.5 µm in this study. Based on the above equation, the relative strength of the ACET effect is strongly related to the AC voltage, and the conductivity/permittivity of sample solutions, which is size-independent. The enrichment process takes only tens of seconds, significantly enhancing both the speed and sensitivity of PFOA detection.

### 3.4. Electrical Characterization of Functionalized Sensor

To enable specific detection of the analyte, effective probe functionalization is crucial for target recognition [29]. In this study, we evaluated surface functionalization following the electrodeposition of a molecularly imprinted polymer (MIP) on the Au interdigitated microelectrode (Au-IDME) by examining the interfacial electrical changes at the electrode surface. Interfacial impedance and capacitance measuring were performed in a 0.1× PBS solution across a frequency range of 100 Hz to 1 MHz during various stages of sensor preparation, with a constant applied voltage of 10 mV. This is easy and superior to most of the methods currently used to characterize the sensor’s surface functionalization [30]. The electrodeposited MIP layer was confirmed by a noticeable increase in the impedance (Figure 2b). Correspondingly, a notable decrease in capacitance was observed, attributed to the added dielectric properties of the polymer layer (Figure 2c). These changes were related to the formation of a thicker polymer film on the electrode surface, which contributed to the reduction in interfacial capacitance C_int_ (as per Equation (1)). Subsequently, when the PFOA molecule was removed during the rising step, the exposure of the imprinted cavities facilitated more efficient ion transport toward the electrode surface, resulting in a marked decrease in impedance. The interaction between PFOA and the MIP-modified surface led to significant alterations at the electrode–electrolyte interface, which contributed to a measurable recovery in the impedance spectrum, alongside a slight decrease in capacitance. These observations confirm the effective functionalization of the Au-IDME surface with the MIP film.

### 3.5. Dose–Response Curve

**Figure 3:**
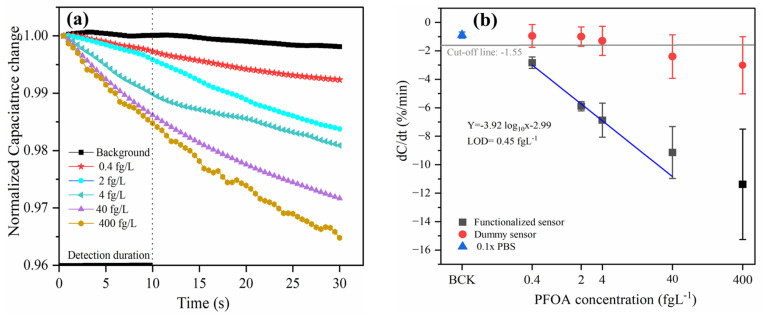
(**a**) Normalized capacitances change as a function of time within 30 s for different levels of PFOA. (**b**) Capacitance change rates in response to different concentrations of PFOA, at 3 kHz and 100 mV.

Under optimized conditions (3 kHz and 100 mV), the sensitivity and dynamic range of the MIP-based capacitive sensor were assessed for detecting PFOA. Initially, we monitored the typical transient curves of normalized capacitance over a 30 s period, revealing clearly distinguishable curves corresponding to different PFOA concentrations in 0.1× PBS (Figure 3a). However, the curves began to overlap at a PFOA concentration of 400 fg/L, signifying the maximum adsorption capacity on the MIP-Au IDME surface, which resulted in a larger standard deviation at this concentration.

These findings demonstrate that a 10 s test duration is sufficient to elicit a significant response from the sensor. The integration of ACET enrichment with the imprinted polymer sensing layer significantly enhances the mass transfer of PFOA to the binding sites. This combination not only improves detection sensitivity but also reduces the time from sample to result, making the sensor efficient for practical applications.

The dose–response curve was established using the response, dC/dt (%/min), defined as the change in normalized capacitance (Figure 3b) and calculated using the least squares method. The calibration equation is given by y (%) = −2.99 − 3.92 log x (fg/L) for the concentration range of 0.4–40 fg/L, demonstrating a strong semi-log linear response with a correlation coefficient (R^2^) of 0.989. The calculated limit of detection (LOD) was found to be 0.45 fg/L, based on the three standard deviations from the background line (analytical buffer, 0.1× PBS). For each data point, at least three sensors were tested under the same condition to obtain the average response and the standard deviation.

To further validate specific detection by the MIP, we employed “dummy sensors”, referring to NIP sensors, for PFOA detection. The responses from these dummy sensors were minimal and showed little correlation with PFOA concentration. This lack of response can be attributed to the nonspecific adsorption of PFOA onto poly-o-phenylenediamine, reinforcing the selectivity of the MIP-based sensor for the target analyte.

### 3.6. Selectivity Study

To evaluate the performance of the MIP-based capacitive sensor, we investigated its selectivity toward various PFAS compounds. Assessing the sensor’s response to structurally similar substances is crucial for determining its specificity and effectiveness [31]. Specifically, we examined the sensor’s response to structurally similar carboxylates, including perfluoroheptanoic acid (PFHA) and perfluoropentanoic acid (PFPA), which differ in carbon chain lengths. We also evaluated long-chain fatty acids, such as octanoic acid (OA), which do not contain C-F bonds in their structure. Additionally, the influence of perfluorooctane sulfonate (PFOS), which shares the same fluorocarbon chain length (C8) as PFOA but has a different head group, was tested to further verify the selectivity of the MIP-based sensor.

**Figure 4:**
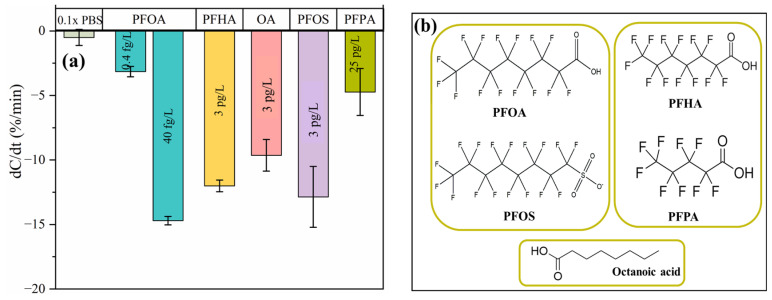
(**a**). MIP-AuIDME in the presence of the other PFAS compounds with similar head and alkyl groups. (**b**) The structure of the PFAS compounds studied.

PFHA, PFOS, and OS were tested at a concentration of 3.0 pg/L, while PFPA was tested at 25 pg/L. Their responses were compared to those of 0.4 fg/L and 40 fg/L PFOA, as shown in Figure 4a. The sensor exhibits a very low response to the shorter-chain perfluorinated compound PFPA at 25 pg/L. In contrast, it shows significant responses to PFOS and PFHA, which have long fluorocarbon chains, at 3.0 pg/L—approximately 70–80% of the response observed for 40 fg/L PFOA. These findings underscore the sensor’s ability to specifically detect PFOA, achieving a selectivity ratio of around 1:100 for long fluorocarbon chains, highlighting its effectiveness in identifying target analytes in complex mixtures. Furthermore, the dC/dt values for 3 pg/L of fluorine-free compounds (OA) are substantial when compared to the value of 40 fg/L for PFOA, emphasizing the critical role of carboxylate head groups tailored during polymerization to form specific binding sites for PFOA molecules.

The results that are summarized in Figure 4a, along with several representative structures of the compounds that were tested (Figure 4b), indicate the highly selective response for PFOA, which is attributed to the selectivity of the MIP toward PFOA.

### 3.7. Imprinting Factor Investigation

The imprinting factor (IF) characterizes the interaction strength between the im-printed polymer towards the template molecule, serving as evidence of the MIP’s selectivity for the target molecule, PFOA. It provides a straightforward estimation of the imprinting effect, which is described as the ratio of normalized capacitance change rate (dc/dt (%/min)) _MIP_ to dc/dt (%/min) _NIP_ [32,33].

The imprinting factor calculated for this study is summarized in Table 2. Relatively high imprinting factors for the analyte of interest implies an acceptable selectivity for the sensor.

## 4. Conclusions

In summary, the developed sensor demonstrated extremely low detection limits for PFAS compounds. This sensing method achieves the highest sensitivity reported so far (Table 1), demonstrating the potential for real-time point-of-use PFAS measurements for aqueous samples. Looking ahead, we aim to advance the commercialization of this sensor, focusing on its application in surface water monitoring and making the sensors more cost-effective. By leveraging its unique capabilities, we seek to address the increasing demand for accurate and reliable PFAS detection in real-world environmental settings, thereby safeguarding both human health and the integrity of ecosystems.

## Figures and Tables

**Table 1:** Comparative representation of different PFOA detection using MIP-based sensors.

Method	Sensor Design	LOD (ngL^−1^)	Dynamic Range (ngL^−1^)	Assay Time	Ref.
DPV ^1^	Au/Cu_2_O@C@NiCo_2_O_4_/MIP	19.46	207–4140	15 min	[12]
Heat transfer	MIP-poly acrylamide	8.0	40–2 × 10^5^	NM ^2^	[13]
Photoelectrochemistry	AgI–BiOINFs ^3^/poly acrylamide	10	20–10^6^	20 min	[14]
Fluorescence	CdTe@CdS-based/poly APTES ^4^	10^4^	10^5^–6 × 10^6^	5 min	[15]
SPR ^5^	Poly VBTe ^6^ and PFDA ^7^	0.81	1–750	NM	[16]
ECL ^8^	GCN ^9^ nanosheets/poly pyrrole	10	20–4 × 10^5^	NM	[17]
Capacitance	MIP/Au-IDMEs/poly o-PD ^10^	4.5 × 10^−7^	4 × 10^−7^–4 × 10^−5^	10 s	This work

^1^ Differential pulse voltammetry; ^2^ not mentioned; ^3^ AgI nanoparticles–BiOI nanoflake array; ^4^ 3-aminopropyltriethoxysilane; ^5^ surface plasmon resonance; ^6^ vinylbenzyl trimethylammonium chloride; ^7^ 1H,1H,2H,2H-perfluorodecyl acrylate; ^8^ electrochemiluminescence; ^9^ graphitic carbon nitride; ^10^ phenylenediamine.

**Table 2:** Imprinting factor of MIP at different concentrations of template.

PFOA Concentration	dc/dt_MIP_	dc/dt_NIP_	IF
0.4 (fg/L)	−2.83	−0.95	2.98
2 (fg/L)	−5.89	−1	5.9
4 (fg/L)	−6.87	−1.3	5.3

## Data Availability

The original contributions presented in this study are included in the article. Further inquiries can be directed to the corresponding author.
